# Changes in the Structure of Amorphous Alloys under Deformation by High-Pressure Torsion and Multiple Rolling

**DOI:** 10.3390/ma16031321

**Published:** 2023-02-03

**Authors:** Galina Abrosimova, Dmitry Gunderov, Evgenia Postnova, Alexandr Aronin

**Affiliations:** 1 Osipyan Institute of Solid State Physics RAS, 142432 Chernogolovka, Russia; 2 Ufa Federal Research Center RAS, Institute of Molecule and Crystal Physics, 450075 Ufa, Russia

**Keywords:** amorphous alloys, deformation, shear bands, free volume

## Abstract

X-ray diffraction and scanning electron microscopy were used to study changes in the structure of amorphous alloys under deformation by high-pressure torsion and multiple rolling. The change in mean nearest neighbor distance (the radius of the first coordination sphere) under deformation was determined. During deformation, shear bands are formed in amorphous alloys, which are regions of lower density compared to the surrounding undeformed amorphous matrix. Shear bands are zones of increased free volume, in which crystallization processes are facilitated. The change in the proportion of free volume under deformation of various types was estimated. The formation of shear bands leads to the appearance of steps on the surface of the samples. The number of shear bands and the surface morphology of deformed amorphous alloys were determined by the type of deformation and the physical properties of the material. The results obtained are discussed within the concept of free volume in the amorphous phase.

## 1. Introduction

Amorphous metal alloys have an unusual structure for alloys and a number of excellent physical properties [1,2]. They can also serve as a precursor for creating a composite amorphous–nanocrystalline structure. The structure of amorphous materials, its dependence on the conditions of preparation, and the effect of heat treatment and deformation on the evolution of an amorphous structure and the formation of nanocrystals in it have been the subject of many studies [3,4,5,6,7,8,9,10]. When an amorphous–nanocrystalline structure is formed, a lot of properties improve [11,12]. The parameters of the nanostructure formed during the crystallization of the amorphous phase depend significantly on the conditions for nanocrystal formation. When nanocrystals are formed during heat treatment, their size turns out to be larger than when they are formed during deformation. Moreover, in the first case, the fraction of nanocrystals turns out to be smaller [13,14]. To elucidate the reasons for this difference, it is necessary to study changes in the structure of the amorphous phase that occur during heating and deformation. It was previously shown that heat treatment could change the type of short-range order in the amorphous phase [15,16,17,18], the formation of ordered regions [19,20,21], the redistribution of one or more components [22,23,24,25], and the formation of a heterogeneous amorphous structure. The heterogeneous amorphous structure formed during heat treatment is due to the inhomogeneous distribution of alloy components. These changes can also occur during the deformation of amorphous alloys [26,27,28,29,30]; however, the most significant changes in the structure are due to the formation of shear bands. Previously, it was found that deformation in amorphous alloys occurred through strongly localized plastic strain in shear bands [31,32,33,34,35,36,37,38]. The thickness of shear bands was 5–20 nm [36,37]. Deformation often results in the branching of shear bands [33]. However, shear bands can be noticeably thicker, and structural changes can propagate up to 200 nm from the shear bands [27,39]. In fact, this means that the deformation of amorphous alloys also results in a heterogeneous amorphous structure [35,36,40]. During deformation, rejuvenation of the amorphous structure is also possible [41,42,43]. On the surface of the sample, shear bands appear in the form of steps, which are the places where shear bands emerge on the surface. It was found that an increase in the degree of deformation led to an increase in surface roughness, an increase in the height of steps, and, under certain conditions, the formation of nanocrystals [44].

It is known that the structure of shear bands is characterized by enhanced free volume content [34,37,40,41,45] and that the diffusion coefficient is several orders of magnitude higher than that in the surrounding amorphous matrix [45]. The change in the density in shear bands was estimated to be ~10% [24,31]. Under deformation, the crystallization of the amorphous phase begins in shear bands, which is usually associated with the higher mobility of atoms in regions with lower density. Free volume is defined as the enhanced space for the movement of atoms under external actions. A lot of studies [46,47,48,49,50,51,52,53,54,55] have been devoted to investigation of the effect of free volume on the structure and properties of a material, and, at present, the concept of free volume is widely used to explain the various properties of metallic glasses. To determine a change in the amount of free volume under various effects on the structure, the method of X-ray diffraction is used most often [28,49,52]. A shift in the position of the main diffuse maximum in X-ray patterns due to heat treatment or deformation means a change in the mean nearest neighbor distance in the amorphous phase, which characterizes a decrease or increase in the free volume fraction in the structure.

The investigation of structural changes during deformation processing in combination with the analysis of steps on the surface (number, location, and other characteristics) can enable estimation of the proportion of deformed material. This work aimed to study changes in the structure of amorphous alloys during deformation.

## 2. Materials and Methods

The structure of amorphous Al-, Fe-, and Zr-based alloys was studied. These alloys are among the most widely studied amorphous and nanocrystalline materials and belong to different groups of alloys. Amorphous Al-Ni-Y and Al-Ni-La alloys are typical representatives of a group of light high-strength materials from the Al-TM-RE group (TM—transition metal, RE—Y, Yb, La, Gd) [2,6,13,14,21,34,37]; amorphous Fe_77_Si_13_B_10_ alloy is a high-induction amorphous magnetic material that serves as the basis for Finemet-type nanocrystalline materials and belongs to the group of the most widely studied ferromagnetic amorphous alloys [4,11,12,18,36]. Amorphous Zr_62.5_Cu_22.5_Al_10_Fe_5_ alloy refers to the most common alloys that can be obtained as a bulk metallic glass [7,26,27,28,29,38].

The compositions of the amorphous alloys after melt quenching are given below. The composition of the alloys was monitored using local X-ray microanalysis by means of a Zeiss Supra 50VP scanning electron microscope. The accuracy of determining composition for all components, except for boron, was about 0.1%. Samples of the amorphous Zr_62.5_Cu_22.5_Al_10_Fe_5_ alloy were prepared as ribbons and rods (bulk metallic glass, BMG). Ingots of the Zr_62.5_Cu_22.5_Al_10_Fe_5_ alloys were made via arc melting using pure (>99.9%) components in a pure argon atmosphere with a Ti getter. The BMG samples were prepared through quenching into a copper mold. The diameter of the rods was 8 mm. The ribbons of all the studied compositions (Zr-, Al-, and Fe-based alloys) were prepared via melt spinning onto a rapidly rotating wheel. The cooling rate was about 10^6^ K/s. The thickness of the Zr_62.5_Cu_22.5_Al_10_Fe_5_ ribbons was about 50 μm. Ingots of the Al-Ni-La(Y) alloys (6–10 at.% Ni and 2–5 at.% La or Y) were prepared via arc melting in eliminated argon from elemental Al (99.99%) and Ni (99.9%) and the Al_3_La(or Y) (99.7%) compound. Amorphous ribbons with a thickness of about 50 μm and a width of 15 mm were obtained via melt spinning in a helium atmosphere. Alloy ingots with nominal compositions of Fe_77_Si_13_B_10_ were prepared through the arc melting of a mixture of Fe (99.9%), Si (99.9%), and B (99.9%); they were melted three times to homogenization before melt spinning in a high-purity argon atmosphere. The width of the ribbons was 1 cm, and the thickness was about 30 μm. The samples were deformed by high-pressure torsion (HPT) and multiple rolling at room temperature. In the first method (HPT), the specimen was placed between two punches, the lower of which was rotated. The degree of deformation varies along the sample radius. Areas near the edge of the sample (in the periphery) are subjected to the greatest deformation; the center of the sample remains practically non-deformed. During repeated rolling, the near-surface regions of the sample are deformed to the greatest extent, while the regions located far from the surface are deformed to a lesser extent. The value of true deformation under HPT is noticeably greater than under multiple rolling. The BMG samples were cut into disks with a thickness of 0.5 mm and polished before deformation. The deformation was carried out at a pressure of 4–6 GPa at a rate of 1 rotation per minute. Cold rolling of the samples was performed in a VEB Schwermaschinenbau four-roll mill using the multiple rolling technique, with the number of rolling passes being 1–150. The level of true deformation was calculated by Formula (1) [56,57]:

(1) $$e=ln{\left(1+{\left(\frac{\varphi \cdot r}{h}\right)}^{2}\right)}^{0.5}+ln(\frac{h_{0}}{h})$$

where *r* is the radius of a sample, φ is the angle of punch rotation, *h*_0_ is the initial thickness of a sample, and *h* is the thickness of a sample after deformation. The samples were subjected to isothermal annealing in a temperature range of 100–300 °C for different time intervals.

**Figure 1:**
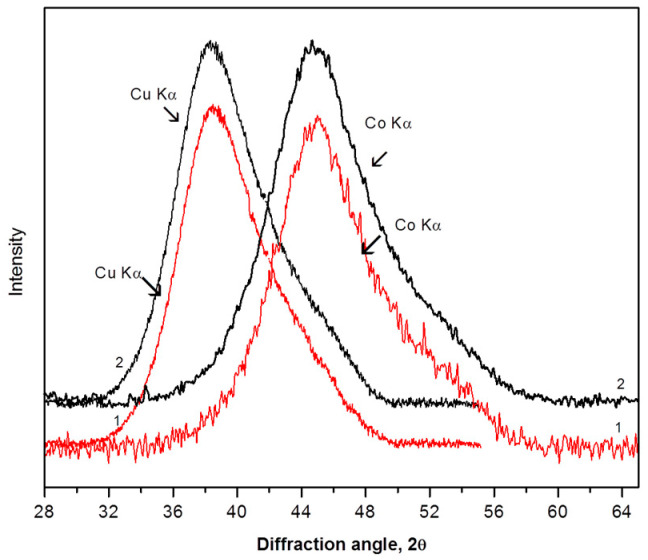
The XRD patterns of amorphous Al_88_Ni_6_Y_6_ alloy after annealing (1) and rolling (2), with alloys obtained using Cu Kα (left curves) and Co Kα (right curves) radiation.

The structure of the samples was investigated by transmission electron microscopy (TEM), scanning electron microscopy (SEM), and X-ray diffraction (XRD) with Cu Kα and Co Kα radiations. Standard computer programs were used for processing the X-ray diffraction spectra (smoothing, background correction, etc.). X-ray diffraction studies were carried out on Siemens D-500 and RigakuSmartLab SE diffractometers, with the scanning step being 0.02°. Initial studies were carried out using Cu Kα radiation (wavelength λ = 1.5406 Å). However, since an important part of the investigation was the analysis of the diffuse peak profile (its width and position), X-ray diffraction studies were also carried out using Co Kα radiation. The wavelength of Co Kα radiation (λ = 1.789 Å) is longer than that of Cu Kα radiation, which allows for “stretching” of the diffuse maximum and the ability to study it more carefully. Figure 1 shows the XRD patterns (region of the first diffuse maximum) for the amorphous Al_88_Ni_6_Y_6_ alloy after annealing (1) and rolling (2), obtained using Cu Kα (left curves) and CoKα (right curves) radiation.

It can be seen that the right maxima are wider than the left ones, and the changes in the positions of peaks 1 and 2 in the right part of the figure are more pronounced. It is easy to calculate that, in the region where the first diffuse maxima exist (in the angular region 30 < 2θ < 60), the change in the corresponding interplanar (or interatomic) spacing is 1.431 Å for Cu Kα radiation and 1.675 Å for Co Kα radiation. This means that the change in the interplanar distance per 1° (angular degree) is 0.056 Å and 0.048 Å for Co Kα and Cu Kα radiation, respectively. This means that under the same collimation conditions, the difference in the accuracy of determining the position of the diffuse maximum when using Co Kα radiation is 17% higher than when using Cu Kα radiation.

To obtain more information about the structure of the amorphous phase, the partial radial distribution functions can be calculated. For a binary alloy A-B, this means the calculation of several functions for the AA, AB, BA, and BB pairs of atoms. The structure factor calculated from the experimental scattering curves for a two-component alloy is the following:

*S*(*Q*) = (1/<*b*>^2^){*c*^2^*_A_b*^2^*_A_S_AA_*(*Q*) + 2 *c_A_ c_B_ b_A_ b_B_ S_AB_*(*Q*) + *c*^2^*_B_b*^2^*_B_S_BB_*(*Q*)} (2)

where *b_A_* and *b_B_* are the amplitudes of coherent scattering of atoms A and B, *c_A_* and *c_B_* are the atomic concentrations of components A and B, *<b> = c_A_b_A_ + c_B_b_B_,* and *Q =* 4*π sin θ/λ* (2*θ* is the scattering angle, λ is the wavelength).

This function is a superposition of the partial functions *S_AA_(Q), S_AB_(Q),* and *S_BB_(Q)* corresponding to the pair correlations of the A-A, A-B, and B-B components. It is clear that to determine the partial structural factors, it is necessary to obtain a system of three equations with different values of *b_A_* or *b_B_*, which requires several independent experiments, such as the use of radiation of different types (X-rays, neutrons, electrons).

There are various ways of defining the total structure factor S(Q), which are called the Faber–Ziman and Bhatia–Thornton formalisms. According to Faber and Ziman [58], partial structure factors describe correlations between the atoms of various chemical elements in the alloy. Formalism of Bhatia and Thornton [59,60] allows for correlations between atoms to be described by the number and sort of neighboring atoms. In fact, partial RDFs describe either correlations between atoms of individual chemical elements (according to Faber–Ziman) or topological short-range order, regardless of the chemical type of an atom (according to Bhatia–Thornton). At present, methods for constructing complete and partial radial distribution functions are not used very often, although it should be noted [61,62,63]. The latter work is especially interesting in that the authors showed the presence of strong chemical ordering in a liquid and amorphous zirconium-based alloy and estimated the stability of the short-range and medium-range order. In the vast majority of cases, when analyzing the structure of metallic glasses, only the radius of the first coordination sphere and its changes under various kinds of influences are estimated. In combination with other research methods, this approach is more productive. Therefore, below we will consider changes in the radius of the first coordination sphere.

The radius of the first coordination sphere, R_1_ (the mean nearest neighbor distance), was determined from the position of the main maximum of the scattering curve using the Ehrenfest equation [64]:

2*R*_1_ *sin θ* = 1.23 *λ* (3)

where *λ* is the wavelength of the radiation used and *θ* is the angular position of the maximum of the scattering curve.

To estimate the proportion of free volume, ΔV, the well-known formula was used:

ΔV = (R_def_^3^ − R_ini_^3^)/R_ini_^3^ × 100% (4)

where R_def_ and R_ini_ are the radii of the first coordination sphere of the deformed and initial (as-prepared) samples, respectively.

## 3. Results and Discussion

**Figure 2:**
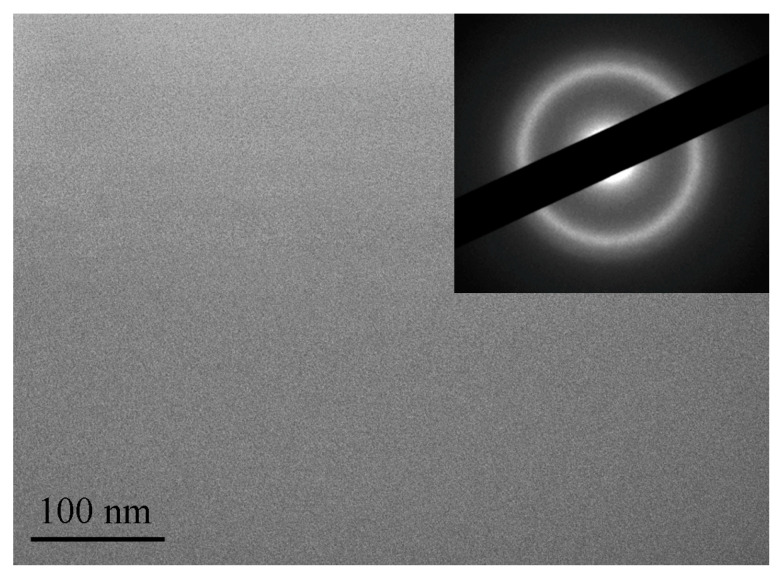
TEM image of the as-prepared bulk amorphous Zr_62.5_Cu_22.5_Al_10_Fe_5_ alloy.

**Figure 3:**
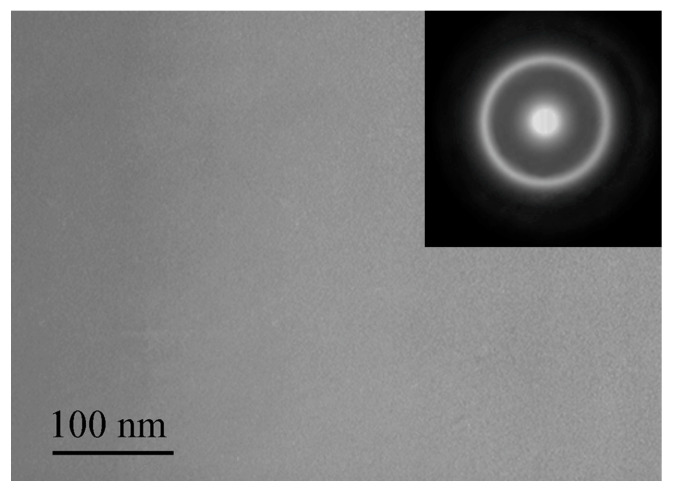
TEM image of the as-prepared amorphous Fe_77_Si_13_B_10_ ribbon.

**Figure 4:**
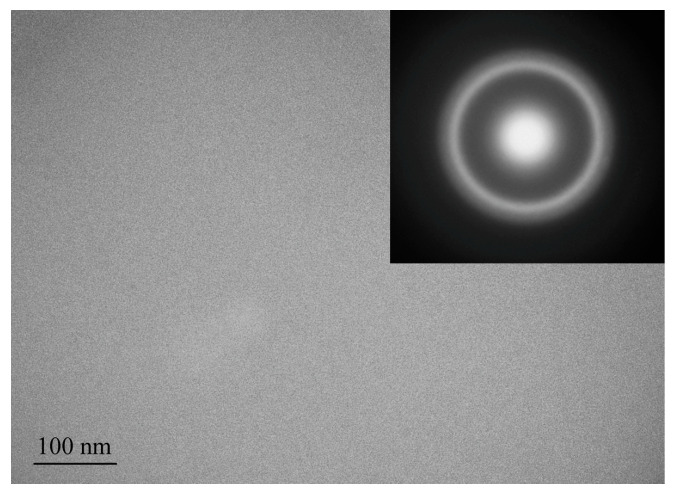
TEM image of the as-prepared amorphous Al_88_Ni_6_Y_6_ ribbon.

After quenching, all alloys were amorphous. Figure 2, Figure 3 and Figure 4 shows TEM images of as-prepared amorphous alloys after quenching. The images and diffraction in the figures do not show any signs of crystalline phases. These data and the presence of only diffuse reflections in the X-ray diffraction patterns (below) make it possible to reliably establish that the structure of the sample is amorphous.

Determination of the mean nearest neighbor distance in the amorphous phase was based on the following measurements. As is known, the prevalence of frequency scattering by the amorphous phase is determined by the following formula [64]:

(5) $$I(S)={NF}^{2}(S)\;\{1+\int_{0}^{\infty }4\pi R^{2}[\rho (R)-\rho_{0}]((\sin SR)/SR)\;dR\}$$

where *N* is the total number of atoms per volume unit, *F*(*S*) is the scattering amplitude, *ρ(R*) is the number of atoms per volume unit located at distance *R* from the selected atom, *ρ*_0_ is the average number of atoms per volume unit, and *S* is the magnitude of the wave vector. The sequence of maxima of the function *I*(*S*) is determined by the sequence of maxima of the function sin (*SR*)/*SR*. This function has peaks at *SR* values of 7.73, 14.06, 20.46, etc. It is easy to determine that the radius of the first coordination sphere (the mean nearest neighbor distance) is the following:

*R*_1_ = 7.73/S_1_ = 14.06/S_2_ = 20.46/S_3_ (6)

This means that the radius of the first coordination sphere can be determined from the value of the wave vector corresponding to any maximum of the scattering intensity curve using Formula (2). Since the first maximum of the scattering curve is the most intense, its position is usually used to determine the radius of the first coordination sphere.

**Figure 5:**
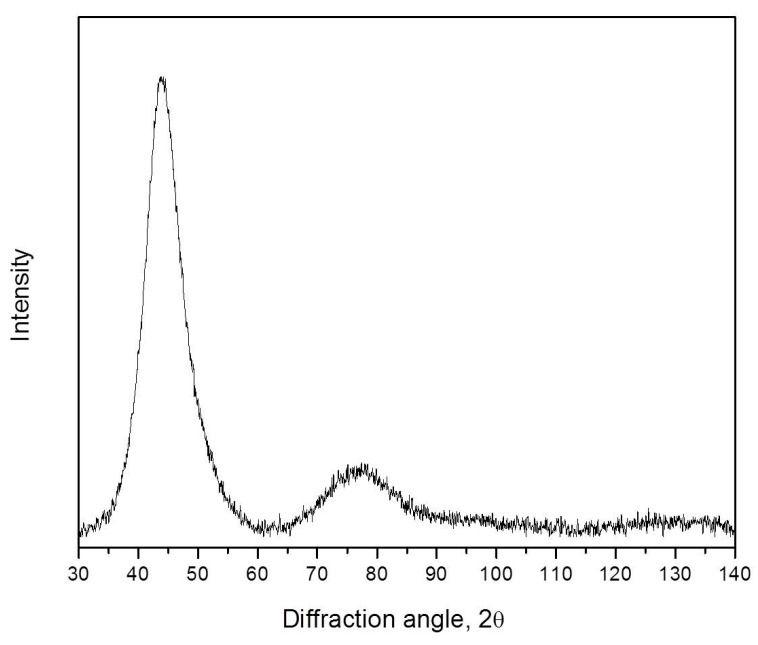
XRD pattern (Co Kα radiation) of the as-prepared bulk amorphous Zr_62.5_Cu_22.5_Al_10_Fe_5_ alloy.

Figure 5 shows an X-ray diffraction pattern of the amorphous Zr_62.5_Cu_22.5_Al_10_Fe_5_ alloy immediately after quenching. It is typical. Below we will analyze the region near the first diffuse maximum.

**Figure 6:**
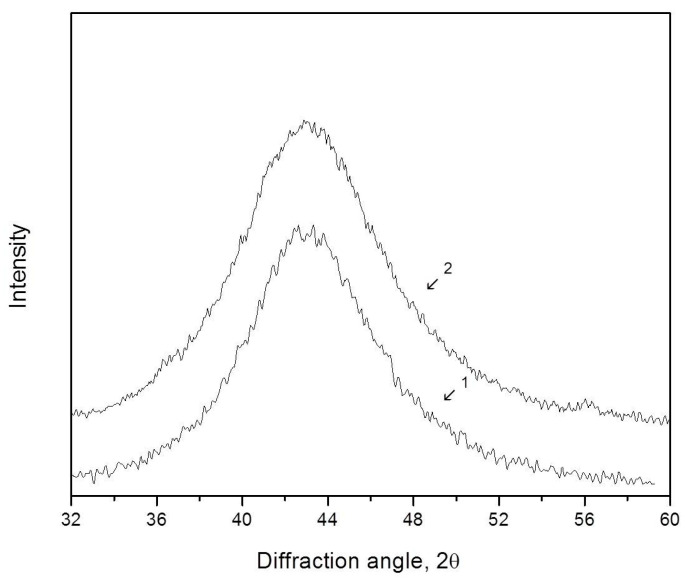
XRD patterns (Co Kα radiation) of the amorphous Zr_62.5_Cu_22.5_Al_10_Fe_5_ BMG before (1) and after (2) deformation by the HPT method.

The radius of the first coordination sphere of the amorphous Zr_62.5_Cu_22.5_Al_10_Fe_5_ alloy determined by Formula (3) is R_1_ = 2.998 Ǻ. After deformation by the high-pressure torsion method (five rotations, true deformation *e* = 4.8), a slight shift of the diffuse maximum to the region of smaller angles is observed. Figure 6 illustrates XRD patterns of the Zr_62.5_Cu_22.5_Al_10_Fe_5_ alloy before (1) and after (2) deformation (the region of the first diffuse maximum). The curves are shifted along the y-axis for clarity. It can be seen that the peak shift is small, but the results are reproducible with repeated surveys. After deformation, the radius of the first coordination sphere is R_1_ = 3.003 Ǻ. The increase in the radius of the first coordination sphere after deformation is ΔR_1_ = 0.005 Ǻ.

Similar results were obtained in the study of samples of the same alloy when prepared as a ribbon. The radius of the first coordination sphere for the ribbons of the amorphous Zr_62.5_Cu_22.5_Al_10_Fe_5_ alloy is the same as for the rods. After deformation by the HPT method (30 rotations), R_1_ = 3.028 Ǻ and the change is ΔR_1_ = 0.025 Ǻ.

**Figure 7:**
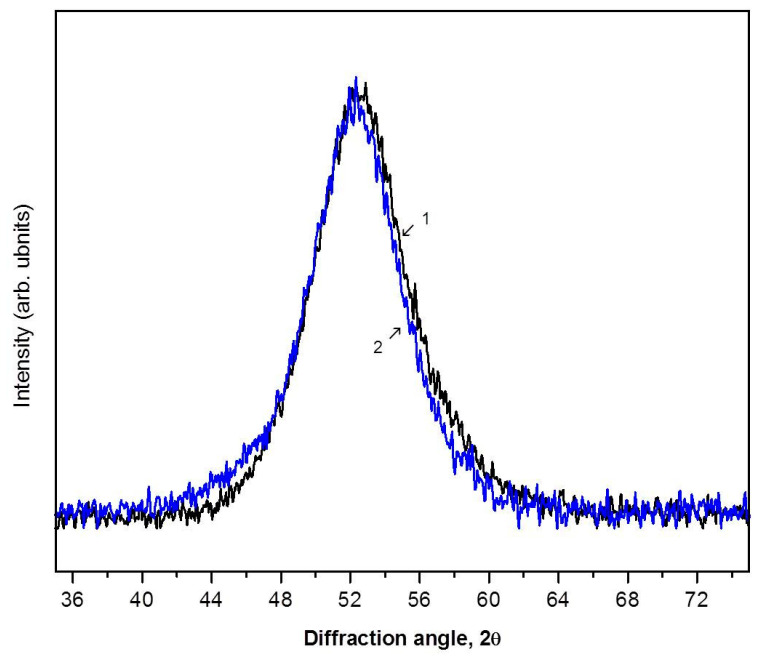
XRD patterns (Co Kα radiation) of the amorphous Fe_77_Si_13_B_10_ ribbon before (1) and after (2) deformation by the HPT method.

Similar results were also obtained in an investigation of amorphous Fe-based alloys. Figure 7 illustrates XRD patterns of the Fe_77_Si_13_B_10_ ribbons before (1) and after (2) deformation (the region of the first diffuse maximum). The values of the radius of the first coordination sphere calculated from the XRD patterns are R_1_ = 2.488 Ǻ for the initial amorphous alloy and R_1_ = 2.492 Ǻ for the deformed sample (4 GPa, one rotation, e = 5.3). Thus, the value calculated from the shift of the diffuse maximum is ΔR_1_ = 0.004 Ǻ. As can be seen from Table 1 (see below), the change in the value of free volume in this alloy is greater than that in the Zr-based alloy, which is consistent with a larger value of true deformation.

An increase in the degree of deformation (by increasing the number of anvil rotations) leads to a greater change in the radius of the first coordination sphere. With an increase in the number of anvil rotations from one to five, the change in the radius of the first coordination sphere in the Fe_77_Si_13_B_10_ alloy increases to ΔR_1_ = 0.007 Ǻ. After this treatment, the samples remain amorphous. Similar results were also observed for bulk Zr-based alloys [29].

Thus, HPT deformation promotes an increase in mean nearest neighbor distance in both bulk amorphous alloys and amorphous ribbons. In the case of the above amorphous Fe_77_Si_13_B_10_ alloy, after rolling, the changes were noticeably smaller and amounted to ΔR_1_ = 0.001 Ǻ.

**Figure 8:**
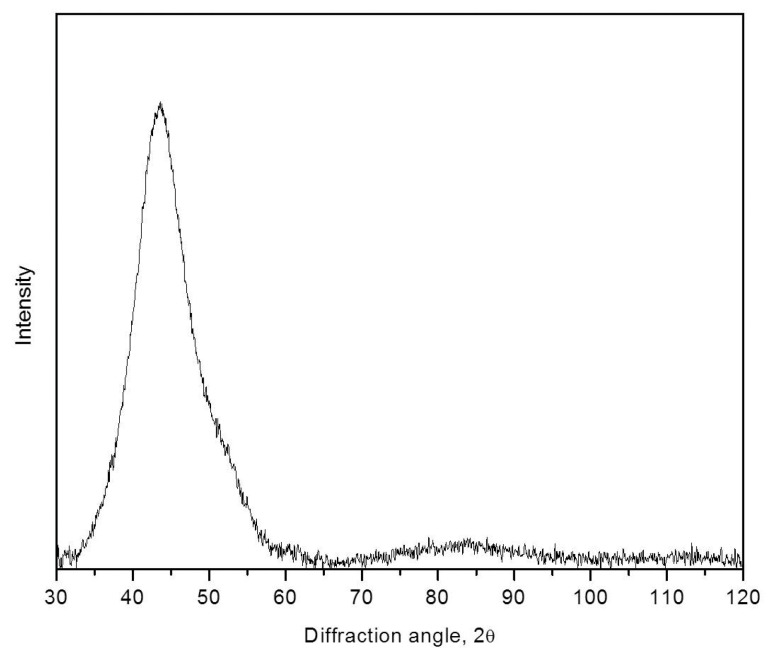
XRD pattern (Co Kα radiation) of the amorphous Al_87_Ni_8_La_5_ ribbon after rolling *(ho-h)/ho/h*_o_ = 35%.

A more complicated situation arises in the study of amorphous Al-based alloys. The issue is related to the fact that, during the deformation of the amorphous phase of Al-TM-RE alloys (TM—transition metal, RE—rare earth metal), amorphous phase decomposition (separation) often occurs and a heterogeneous amorphous structure is formed. This heterogeneous amorphous structure consists of regions enriched or depleted in terms of one of the alloy components. In this case, the observed shift of the diffuse maximum to smaller angles is due to the appearance of amorphous regions enriched in the component with the largest atomic size (in these systems, this is an atom of a rare-earth element). It should be noted that the approach considered here is valid, first of all, for metallic glasses. In a number of oxide glasses or in one-component systems (liquid Sn or Ge); therefore, the origin of peak asymmetry may be more complicated. Figure 8 shows an X-ray diffraction pattern of the Al_87_Ni_8_La_5_ amorphous alloy after rolling (thickness change *(ho-h)/ho* = 35%). It is clearly seen that it is asymmetric and, obviously, is a superposition of two maxima.

**Figure 9:**
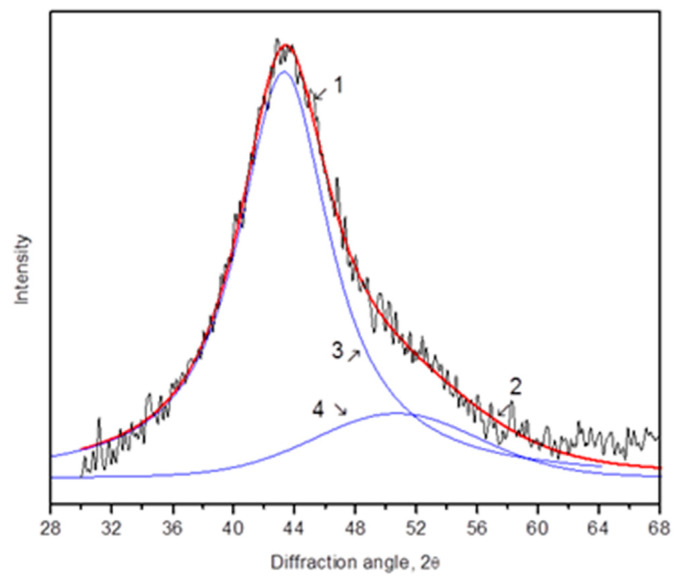
XRD pattern (Co Kα radiation) of the annealed Al_87_Ni_8_La_5_ ribbon (150 °C 15 h) (1—experimental curve, 2—approximation, which is the sum of curves 3 and 4).

Figure 9 depicts an XRD pattern (the region of the first diffuse maximum) of the amorphous Al_87_Ni_8_La_5_ alloy annealed at 150 °C for 5 h. As in the XRD pattern of the rolled sample, the diffuse maximum is asymmetric and is a superposition of two maxima. The different position of the two subpeaks indicates that the sample contains regions that differ in composition. Based on an analysis of the atomic size and composition of metallic glass, it is easy to conclude that the two diffuse maxima correspond to regions enriched and depleted in yttrium (and/or nickel). Curves 3 and 4 in Figure 9 correspond to amorphous phases enriched (3) and depleted (4) in terms of a rare-earth component, and curve 2 is the sum of curves 3 and 4. It can be seen that the total X-ray diffraction pattern (2) is in good agreement with the experimental curve (1).

**Figure 10:**
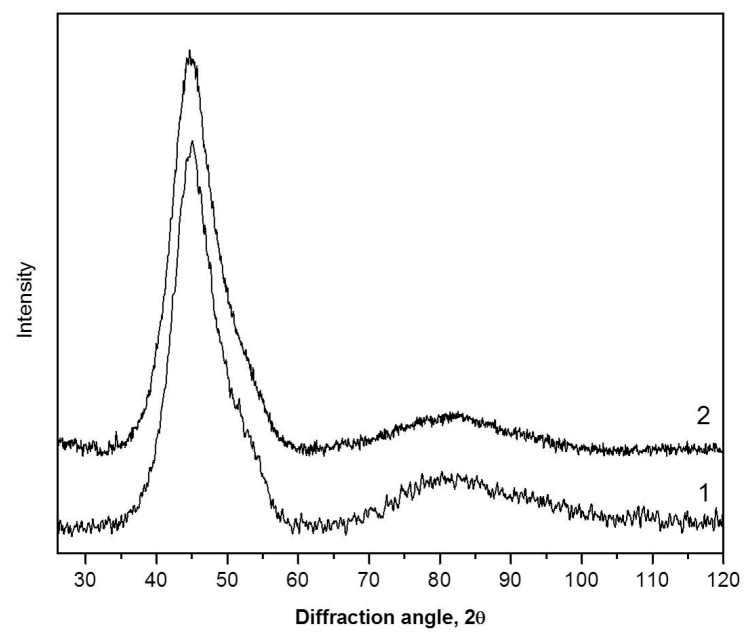
XRD patterns (Co Kα radiation) of the annealed Al_88_Ni_6_Y_6_ ribbon after annealing (1) and rolling (2).

Figure 10 shows XRD patterns of the amorphous Al_88_Ni_6_Y_6_ alloy annealed at 100 °C for 30 h (1) and rolled by 40% (2). For this alloy, the values of ΔR_1_ given in Table 1 correspond to the first (left) sub-peak. It is clearly seen here that both heat treatment and deformation of the amorphous alloy lead to the formation of regions of different chemical composition. Similar changes were observed in a number of other studies [24,65,66,67]. Changes in X-ray diffraction patterns depend on the deformation or annealing conditions and increase with increasing heat treatment time [24] or the degree of deformation. Although electron microscopic studies demonstrate that numerous shear bands are also formed in these alloys under deformation, it is impossible to distinguish their effect correctly. It is important to note that the changes in the radius of the first coordination sphere corresponding to the left subpeak turn out to be much larger than those in Fe- or Zr-based alloys and mainly reflect the formation of a new amorphous phase enriched in a rare-earth component, rather than the contribution of shear bands. The amount of separation can be different; it depends on the type and degree of deformation, temperature, and the duration of annealing. However, in any case, the formation of a heterogeneous structure is a factor that makes it difficult to correctly determine the change in the average distance between atoms due to the free volume.

Using Formula (4) and the values of the radii of the first coordination sphere and their changes, it is possible to determine the change in the free volume in the amorphous structure that results from deformation by the high-pressure torsion (HPT) and multiple rolling (MR) methods. The data obtained are listed in Table 1.

The value of the radius of the first coordination sphere, of course, depends on the size of the alloy atoms, and its change depends on the degree of deformation. It should be noted that a change in the free volume value also depends on the conditions for obtaining the sample. When preparing amorphous alloys using the melt spinning method, the structure of the melt actually freezes. Since the structure and density of the melt depend on temperature, the density of the amorphous phase will also depend on the temperature of the melt before quenching and the cooling rate. The value of the frozen free volume in this case will also be determined by these parameters, as well as by the prehistory of the sample before deformation onset. For example, during aging, structural relaxation processes are possible, leading to a decrease in free volume [68,69,70,71]. Therefore, when studying the effect of external actions on the change in free volume, it is crucial to carry out structural studies immediately before the onset of deformation or heat treatment and immediately after their completion.

As mentioned above, changes in free volume during the deformation of amorphous alloys are due to the formation of shear bands (regions of localization of plastic deformation), which depends on both the degree of deformation and the elastic constants of the material. After deformation, the structure of the sample is actually a nanoglass consisting of amorphous regions with the amorphous phase that did not change under deformation and amorphous regions of reduced density (shear bands). Therefore, the radius of the first coordination sphere (R_1_) determined above is averaged as follows:

R_1_ = (*a R*_1sample_ + *b R*_1shear bands_) (7)

where *a* and *b* are the volume fractions of the unchanged amorphous phase and shear bands, respectively. Since the volume fraction of shear bands is small, the change in the average radius of the first coordination sphere

ΔR_1_ = R_1def_ − R_1ini_ (8)

should be small, which is observed experimentally. For the above sample, this change is ΔR_1_ = 0.008 Ǻ. An estimate of the change in the radius of the first coordination sphere provides a value of ~0.3%. This corresponds to an increase in free volume, ΔV, of about 0.9%, which is consistent with known data regarding changes in the structure of amorphous alloys during HPT [27,37].

It should be emphasized that the data on changes in the radii of coordination spheres and changes in density (or excess free volume, determined by Formula (3)) are average values. The values of coefficients *a* and *b*, which characterize the volume fractions of the unchanged amorphous phase (a) and shear bands (*b*), differ significantly: *a* >> *b*. This means that the changes in the structure in the shear bands are much larger than those observed. It is these large changes that lead to an increase in the diffusion coefficient in these regions of several orders of magnitude, as noted in [36].

**Figure 11:**
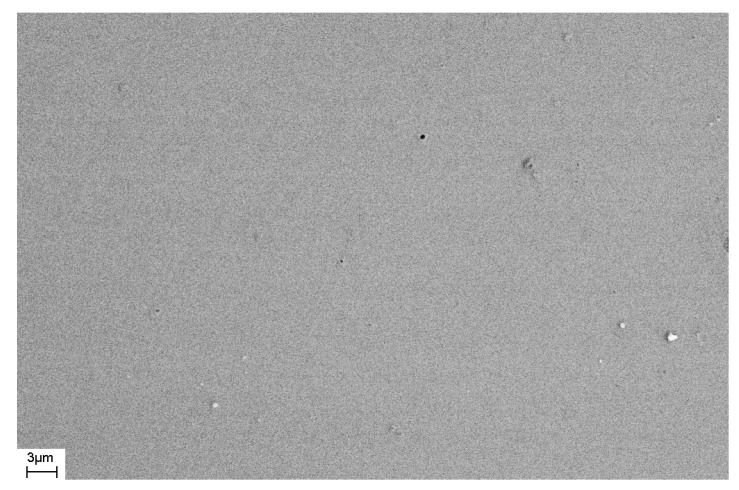
SEM image of the surface of the as-prepared Fe_77_Si_13_B_10_ ribbon.

**Figure 12:**
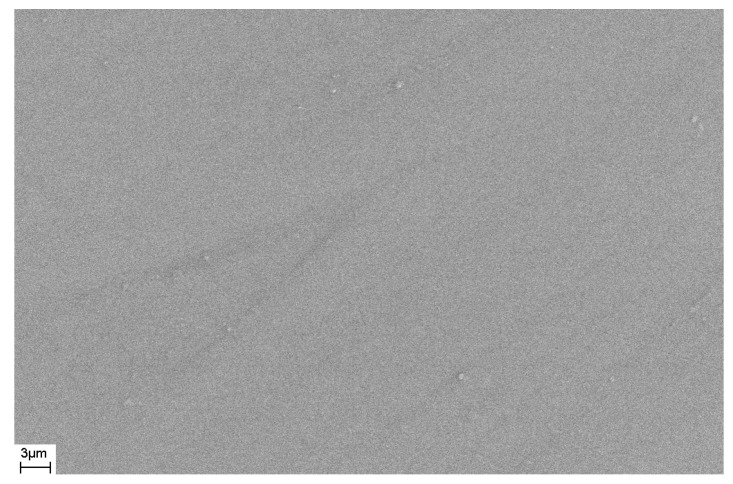
SEM image of the surface of the as-prepared Al_88_Ni_10_Y_2_ ribbon.

**Figure 13:**
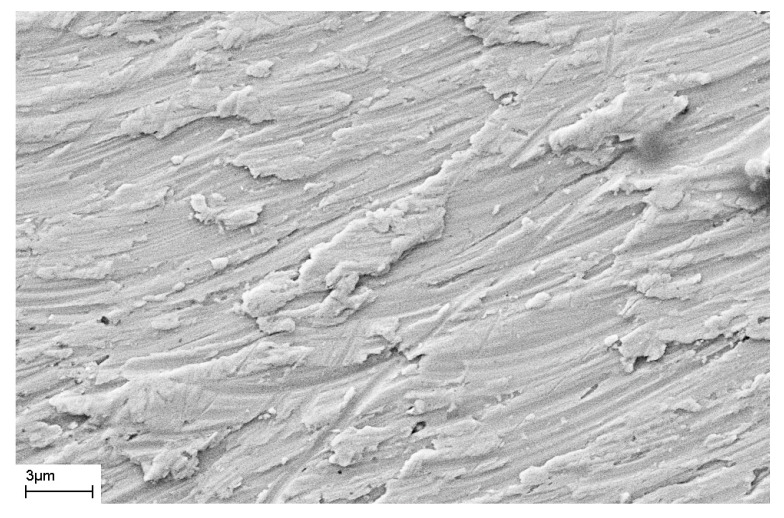
SEM image of the surface of the bulk amorphous Zr_62.5_Cu_22.5_Al_10_Fe_5_ alloy deformed by the HPT method.

**Figure 14:**
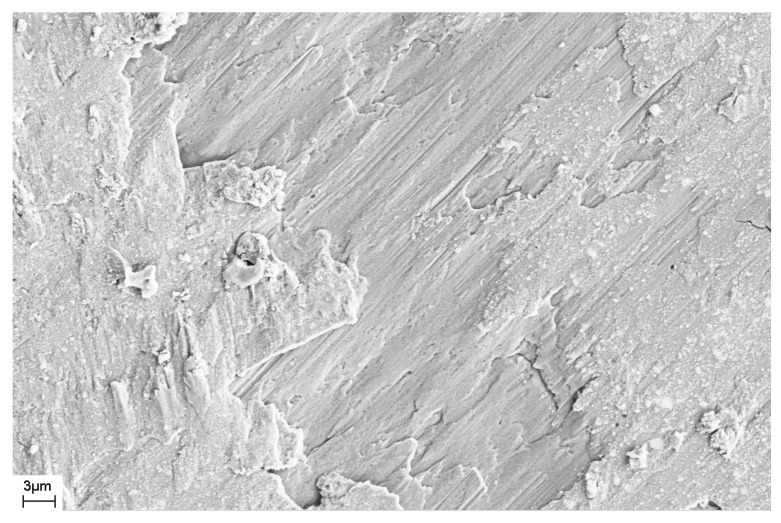
SEM image of the surface of the amorphous Fe_77_Si_13_B_10_ ribbon deformed by the HPT method.

**Figure 15:**
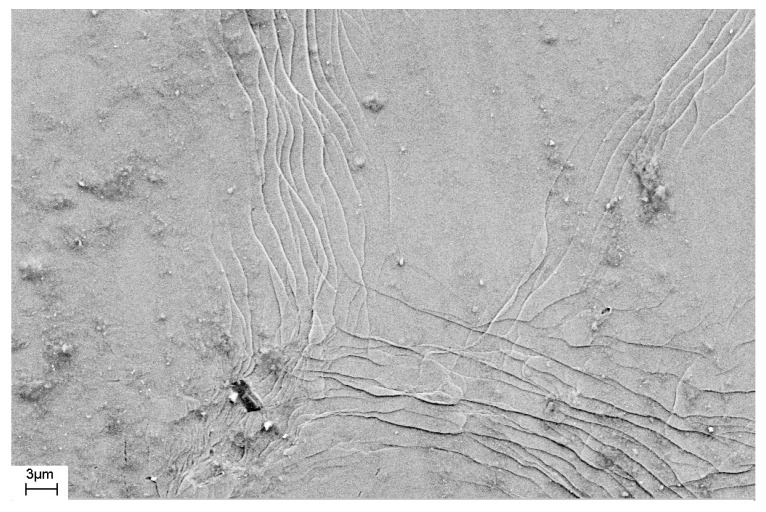
SEM image of the surface of the amorphous Fe_77_Si_13_B_10_ ribbon deformed by rolling (one rolling pass).

**Figure 16:**
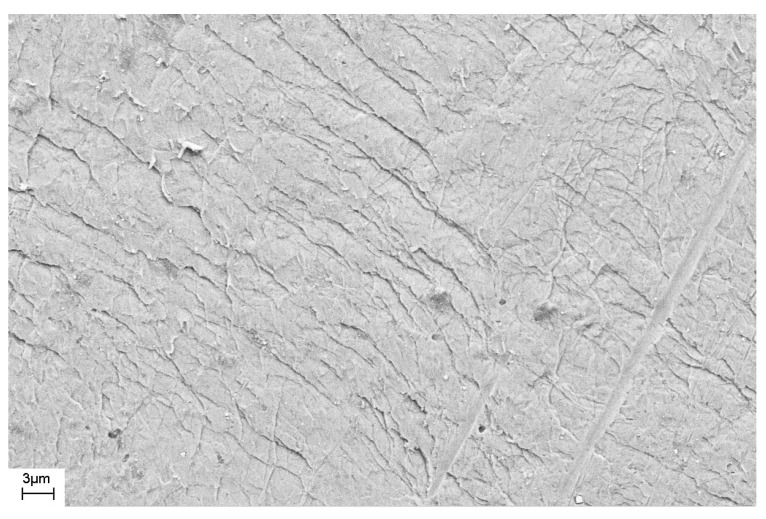
SEM image of the surface of the amorphous Fe_77_Si_13_B_10_ribbon deformed by rolling (*(ho-h)/ho* ≈ 5%).

**Figure 17:**
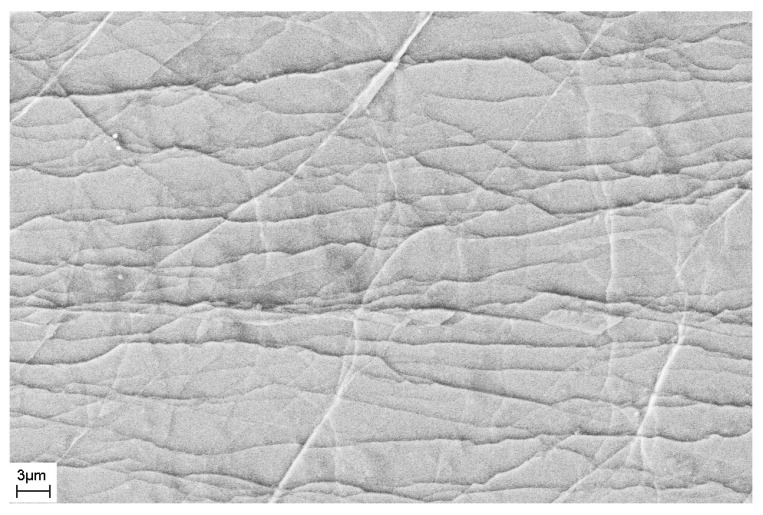
SEM image of the surface of the amorphous Al_88_Ni_10_Y_2_ ribbon deformed by rolling (*(ho-h)/ho* ≈ 50%).

As noted above, during deformation, steps are formed on the surface of the sample that characterize the places where shear bands emerge on the surface of the sample. The number of shear bands, their morphology, and their location also depend on the elastic constants of the material, as well as the type and degree of deformation. Figure 11 and Figure 12 show SEM images of the surfaces of as-prepared amorphous Fe_77_Si_13_B_10_ and Al_88_Ni_10_Y_2_ alloys. The surfaces of the as-prepared alloys are smooth and do not contain any features. Figure 13, Figure 14, Figure 15, Figure 16 and Figure 17 shows the surface structure of the investigated alloys after deformation of different types.

One can see that the surfaces of the deformed samples contain numerous steps corresponding to shear bands. The size and number of steps depend on the type and degree of deformation, as well as on the chemical composition of the alloy. The results of studying the surface morphology of deformed amorphous alloys show that the number of shear bands increases with the degree of deformation, which is indicated by the change in material volume due to deformation. Such an increase in the number of steps with an increase in the degree of deformation is illustrated in Figure 15 and Figure 16. These figures show the surfaces of Fe_77_Si_13_B_10_ samples after rolling. After the first rolling pass, steps are formed on the surface, and their formation occurs unevenly. Thus, Figure 15 shows that some areas of the surface do not contain steps, while in other places the formation of steps is noted. With an increase in the degree of deformation, the number of steps increases, they lose their rectilinear shape, and their multiple intersection with each other is observed (Figure 16). The greater the number of shear bands, the greater, naturally, is the proportion of material with a large first coordination sphere radius value (the mean nearest neighbor distance).

Based on the obtained images of the surface of the Fe_77_Si_13_B_10_ alloy deformed by rolling (Figure 16), the total length of the steps on the surface was estimated. The frame size in which the length of the shear bands was measured was 70.4 μm × 46.4 μm = 3267 μm^2^.

The length of the steps (L) was estimated using direct measurements from the images. It was approximately 2100 μm in the image. If we assume that each step was formed as a result of the shear band coming to the surface, that the shear bands have the same thickness throughout the sample, and that they do not multiply inside the sample, the volume fraction of material (ν) in the shear band can be estimated. The volume fraction of the amorphous phase in the shear bands can be determined from Formula (9):

ν = L ∗ δ/S(9)

where δ is a shear band thickness and S is the image area.

If we assume that the thickness of the shear band is 20 nm [14,16,22], and its density is less than the density of the amorphous matrix by 10% [27,37], then the density of the deformed amorphous sample will be 0.9987 ρ_0_ (ρ_0_ is the density of the undeformed amorphous phase). This means that the change in density is 0.13%, which agrees with the XRD data (Table 1).

In the above calculations it was assumed that the step corresponds to a single 20 nm-thick shear band. At the same time, our studies of the fine structure of the bands using atomic force microscopy [44] show that the steps observed using scanning electron microscopy, in turn, have a complex structure. They are formed as a result of the action of several single shear bands. In addition, the difference in density of the deformed and undeformed amorphous phases may be less than that indicated in [27,37]. For example, in [72], this difference is 1.4%. In this case, the proportion of material in the shear bands may differ.

The results obtained indicate that deformation of the amorphous phase contributes to an increase in the fraction of free volume, which leads to an increase in the average radius of the first coordination sphere (distance between atoms). It manifests itself in XRD patterns as a shift of the diffuse maximum to the region of smaller angles. The change in the proportion of free volume depends on the type and degree of deformation, as well as on the elastic constants of the material. The results obtained are an example of such changes and are not sufficient for an exhaustive analysis of the effect of the degree and type of deformation on the value of free volume and its dependence on the listed parameters. This requires wider research on a large group of amorphous alloys deformed under different conditions.

## 4. Conclusions

Changes in the structure of amorphous Zr_62.5_Cu_22.5_Al_10_Fe_5_, Fe_77_Si_13_B_10_, and Al-Ni-La(Y) alloys prepared via melt quenching in the form of ribbons and/or rods (BMG) and further deformed by the high-pressure torsion and multiple rolling methods were studied. Both methods of deformation lead to the formation of steps on the sample surface due to the formation of shear bands (regions of localized plastic deformation). These shear bands are zones of enhanced free volume (lower density). The formation of shear bands leads to an increase in the average radius of the first coordination sphere of the amorphous phase. The change in mean nearest neighbor distance in an amorphous structure depends on the conditions of the preparation of the material, the type and degree of deformation, and the physical constants of the material (Young’s modulus, shear modulus).

## Figures and Tables

**Table 1:** Changes in the radius of the first coordination sphere (ΔR_1_) and the value of free volume as a result of deformation.

Chemical Composition, Sample Type	Deformation Conditions	ΔR_1_, Ǻ	ΔV = (R_def_^3^ − R_ini_^3^)/R_ini_^3^ × 100%
Zr_62.5_Cu_22.5_Al_10_Fe_5_, BMG	HPT 6 GPa, RT, N = 5	0.005	0.50
Zr_62.5_Cu_22.5_Al_10_Fe_5_, ribbon	HPT 6 GPa, RT, N = 30	0.025	2.51
Fe_77_Si_13_B_10_, ribbon	HPT 4 GPa, RT, N = 1	0.007	0.84
Fe_77_Si_13_B_10_, ribbon	MR, *(ho-h)/ho* = 5%	0.001	0.12
Al_88_Ni_10_Y_2_, ribbon *	MR, *(ho-h)/ho* = 50%	0.010	1.05
Al_87_Ni_8_Y_5_, ribbon *	MR, *(ho-h)/ho* = 35%	0.023	[24]
Al_87_Ni_8_La_5_, ribbon *	Heat treatment, 150 °C 15 h	0.550	[24]

* Accompanied by the formation of a heterogeneous amorphous structure.

## Data Availability

The data can be provided upon request.
